# 1-Methyl­piperazine-1,4-diium dipicrate

**DOI:** 10.1107/S1600536811001024

**Published:** 2011-01-15

**Authors:** Grzegorz Dutkiewicz, S. Samshuddin, B. Narayana, H. S. Yathirajan, Maciej Kubicki

**Affiliations:** a Department of Chemistry, Adam Mickiewicz University, Grunwaldzka 6, 60-780 Poznań, Poland; b Department of Studies in Chemistry, Mangalore University, Mangalagangotri 574 199, India; c Department of Studies in Chemistry, University of Mysore, Manasagangotri, Mysore 570 006, India

## Abstract

In the crystal structure of the title compound [systematic name: 1-methyl­piperazine-1,4-diium bis­(2,4,6-trinitro­phen­ol­ate)], C_5_H_14_N_2_^2+^·2C_6_H_2_N_3_O_7_^−^, the ionic components are connected by relatively strong N—H⋯O hydrogen bonds into centrosymmetric six-membered conglomerates, which comprise two dications and four anions. Besides Coulombic inter­actions, only weak C—H⋯O inter­actions and some stacking between picrates  (separation between the planes of *ca*. 3.4 Å but only a small overlapping) can be identified between these ‘building blocks’ of the crystal structure. The piperazine ring adopts a chair conformation with the methyl substituent in the equatorial position. In the picrate anions, the twist angles of the nitro groups depend on their positions relative to the phenolate O atom: it is much smaller for the NO_2_ groups *para* to the C—O^−^ group [15.23 (9)and 3.92 (14)°] than for the groups in the *ortho* positions [28.76 (13)–39.84 (11)°].

## Related literature

For examples of the biological activity of piperazines: Brockunier *et al.* (2004 ▶); Bogatcheva *et al.* (2006 ▶). For the crystal structures of simple piperidinium picrates, see: Fun *et al.* (2010 ▶); Li *et al.* (2009 ▶); Verdonk *et al.* (1997 ▶); Wang & Jia (2008 ▶). For a description of the Cambridge Structural Database, see: Allen (2002 ▶). For asymmetry parameters, see: Duax & Norton (1975 ▶). 
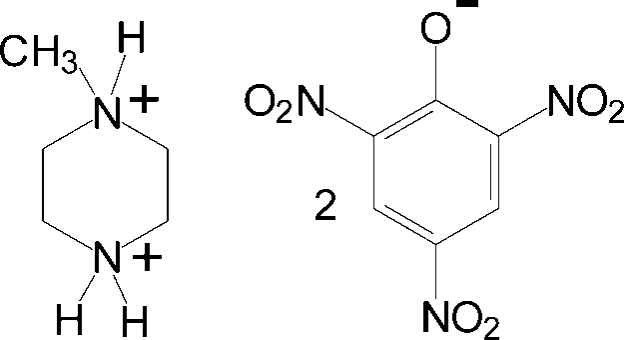


## Experimental

### 

#### Crystal data

C_5_H_14_N_2_^2+^·2C_6_H_2_N_3_O_7_^−^
*M* *_r_* = 558.39
Triclinic, 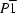

*a* = 8.2001 (12) Å
*b* = 10.1780 (15) Å
*c* = 13.7399 (18) Å
α = 89.798 (12)°
β = 78.130 (11)°
γ = 81.558 (12)°
*V* = 1109.6 (3) Å^3^
*Z* = 2
Mo *K*α radiation
μ = 0.15 mm^−1^
*T* = 295 K
0.4 × 0.15 × 0.07 mm

#### Data collection

Oxford Diffraction Xcalibur Eos diffractometer
Absorption correction: multi-scan (*CrysAlis PRO*; Oxford Diffraction, 2009 ▶) *T*_min_ = 0.936, *T*_max_ = 1.000
21056 measured reflections
4891 independent reflections
3624 reflections with *I* > 2σ(*I*)
*R*_int_ = 0.021

#### Refinement

*R*[*F* ^2^ > 2σ(*F* ^2^)] = 0.044
*wR*(*F* ^2^) = 0.123
*S* = 0.95
4891 reflections
424 parameters
H atoms treated by a mixture of independent and constrained refinement
Δρ_max_ = 0.24 e Å^−3^
Δρ_min_ = −0.30 e Å^−3^

### 

Data collection: *CrysAlis PRO* (Oxford Diffraction, 2009 ▶); cell refinement: *CrysAlis PRO*; data reduction: *CrysAlis PRO*; program(s) used to solve structure: *SIR92* (Altomare *et al.*, 1993 ▶); program(s) used to refine structure: *SHELXL97* (Sheldrick, 2008 ▶); molecular graphics: *Stereochemical Workstation Operation Manual* (Siemens, 1989 ▶); software used to prepare material for publication: *SHELXL97*.

## Supplementary Material

Crystal structure: contains datablocks I, global. DOI: 10.1107/S1600536811001024/fl2330sup1.cif

Structure factors: contains datablocks I. DOI: 10.1107/S1600536811001024/fl2330Isup2.hkl

Additional supplementary materials:  crystallographic information; 3D view; checkCIF report

## Figures and Tables

**Table 1:** Hydrogen-bond geometry (Å, °)

*D*—H⋯*A*	*D*—H	H⋯*A*	*D*⋯*A*	*D*—H⋯*A*
N11—H11⋯O1*A*	0.892 (18)	1.831 (18)	2.6305 (17)	148.1 (16)
N11—H11⋯O22*A*	0.892 (18)	2.356 (18)	2.996 (2)	128.8 (14)
N14—H14*B*⋯O1*B*^i^	0.88 (2)	1.98 (2)	2.8181 (19)	157.3 (17)
N14—H14*A*⋯O1*B*	0.92 (2)	1.99 (2)	2.7962 (18)	146.4 (18)
N14—H14*A*⋯O22*B*	0.92 (2)	2.28 (2)	2.992 (2)	133.9 (16)
C5*A*—H5*A*⋯O21*A*^ii^	0.917 (19)	2.476 (19)	3.383 (2)	170.3 (16)
C5*B*—H5*B*⋯O21*B*^iii^	0.913 (18)	2.487 (18)	3.394 (2)	172.3 (15)
C11*A*—H11*C*⋯O41*A*^iv^	0.93 (3)	2.48 (3)	3.345 (2)	155 (2)
C11*A*—H11*A*⋯O62*A*^iii^	0.94 (3)	2.57 (3)	3.496 (3)	168 (2)
C13—H13*A*⋯O62*B*^v^	0.96 (2)	2.46 (2)	3.386 (2)	162.9 (17)

Symmetry codes: (i) 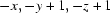; (ii) 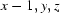; (iii) 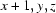; (iv) 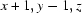; (v) 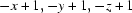.

**Fig. 1.**
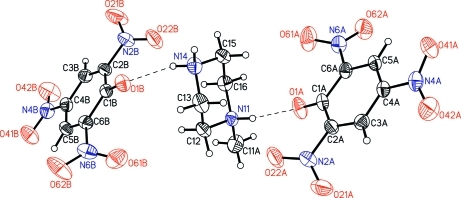
Anisotropic ellipsoid representation of the ionic components of I together with atom labelling scheme. The ellipsoids are drawn at 50% probability level, hydrogen atoms are depicted as spheres with arbitrary radii; hydrogen bonds are shown as dashed lines.

**Fig. 2.**
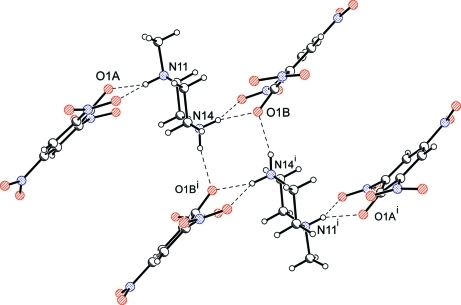
The centrosymmetric dimer of salt I; hydrogen bonds are shown as dashed lines. Symmetry codes: (i) -x,1 - y,1 - z.

**Fig. 3.**
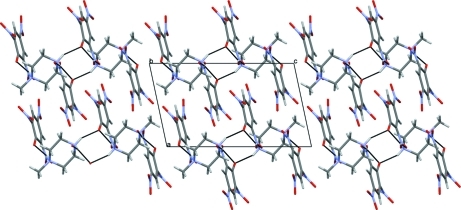
The crystal packing as seen approximately along y-direction. Hydrogen bonds are shown as dashed lines.

## supplementary crystallographic information

### Comment

Piperazines are among the most important building blocks in today's drug discovery. They are found in biologically active compounds across a number of different therapeutic areas such as antifungal, antibacterial, antimalarial, antipsychotic, antidepressant and antitumour activity against colon, prostate, breast, lung and leukemia tumors (for instance, Brockunier *et al.*, 2004, Bogatcheva *et al.*, 2006). A small number of piperazinium picrates or piperazinediium dipicrates have been structurally characterized, however generally the cations were heavily substituted. On the other hand, picric acid (p*K*_a_=0.38) has been studied for its ability to form salts which display wide spectrum of intermolecular interactions, for instance hydrogen bonds of different strengths and/or π···π stacking interactions. In the course of our studies of picrates of simple organic cations we have determined the crystal and molecular structure of the title compound (I: 1-methylpiprazinediium di(2,4,6-trinitrophenolate), Scheme 1).

In the CSD (Allen, 2002; Version 5.31 of Nov. 2009, updated August 2010) there are only a few picrates of simple piperazinium derivatives, for instance 4-(4-carboxybenzyl)-1-methylpiperazin-1-ium picrate (Li *et al.*, 2009), 1-(2-methoxyphenyl)piperazinium picrate (Verdonk *et al.*, 1997) or piperazine-1,4-diium–dipicrate piperazine complex (Wang & Jia, 2008). Also some more complicated structures were reported, for instance 4-(3-Carboxy-1-ethyl-6-fluoro-4-oxo-1,4- dihydro-7-quinolyl)-1-methylpiperazinium picrate (Fun *et al.*, 2010).

In the crystal structure I there are two picrate anions and 1-methylpiperidinediium dication (Fig. 1); the presence of ionic species is supported by the successful location and refinement of the hydrogen atoms at both nitrogen atoms in the piperidine ring as well as by inspection of the pattern of bond distances and angles. The piperazine ring adopts an almost ideal chair conformation; the values of asymmetry parameters (Duax & Norton, 1975), which measure the deviations from the ideal symmetry (in the case *D_3_**_d_*), are very small, less than 1.6°. The methyl substituent is in the equatorial position as can be seen from the torsion angles C13—C12—C11—C11A: 176.60 (15)° and C15—C16—C11—C11A: -176.72 (14)°. Both aromatic rings are in a good approximation planar, maximum deviation from the least-squares plane calculated by the six ring atoms is 0.0248 (11)Å in the anion A and 0.0297 (10)Å in anion B. The nitro groups are twisted with respect to the ring planes, for the groups *ortho* with respect to the C—O^-^ group (at C2 and C6) this twist is of course significantly larger (ranging from 28.76 (13)° to 39.84 (11)°) than for the groups in *para* positions, at C4 (15.23 (9)° in anion A, only 3.92 (14)° in B).

In the crystal structure the building block is made up of a centrosymetric pair of hydrogen bonded ionic components: two dications and four anions (Table 1, Fig. 2). Using graph set notation one can identify - taking into account the primary interactions only - the centrosymmetric ring *R*^2^_4_(8) and dimeric D motifs. Interestingly no strong hydrogen bonds are observed between these structures; besides the coulombic interactions only weak C—H···O and some stacking between picrates (Fig. 3) organize the crystal packing.

### Experimental

1-Methyl piperazine (1.00 g, 0.01 mol) was dissolved in 20 ml of alcohol. Picric acid (4.58 g, 0.02 mol) was dissolved in 50 ml of water. Both the solutions were mixed and to this, 5 ml of 3*M* HCl was added and stirred for few minutes. The formed complex was filtered and dried, crystals appropriate for X-ray data collection were found without further recrystallization (m. p. >523 K). Composition: Found (Calculated): C: 36.48 (36.57); H: 3.20 (3.25); N:19.98 (20.07).

### Refinement

Hydrogen atoms were located in difference Fourier maps and isotropically refined.

### Crystal data

**Table:**

C_5_H_14_N_2_^2+^·2C_6_H_2_N_3_O_7_^−^	*Z* = 2
*M**_r_* = 558.39	*F*(000) = 576
Triclinic, *P*1	*D*_x_ = 1.671 Mg m^−^^3^
Hall symbol: -P 1	Mo *K*α radiation, λ = 0.71073 Å
*a* = 8.2001 (12) Å	Cell parameters from 12041 reflections
*b* = 10.1780 (15) Å	θ = 3.0–28.0°
*c* = 13.7399 (18) Å	µ = 0.15 mm^−^^1^
α = 89.798 (12)°	*T* = 295 K
β = 78.130 (11)°	Block, yellow
γ = 81.558 (12)°	0.4 × 0.15 × 0.07 mm
*V* = 1109.6 (3) Å^3^	

### Data collection

**Table:**

Oxford Diffraction Xcalibur Eos diffractometer	4891 independent reflections
Radiation source: Enhance (Mo) X-ray Source	3624 reflections with *I* > 2σ(*I*)
graphite	*R*_int_ = 0.021
Detector resolution: 16.1544 pixels mm^-1^	θ_max_ = 28.0°, θ_min_ = 3.0°
ω scans	*h* = −10→10
Absorption correction: multi-scan (*CrysAlis PRO*; Oxford Diffraction, 2009)	*k* = −13→12
*T*_min_ = 0.936, *T*_max_ = 1.000	*l* = −18→18
21056 measured reflections	

### Refinement

**Table:**

Refinement on *F*^2^	Primary atom site location: structure-invariant direct methods
Least-squares matrix: full	Secondary atom site location: difference Fourier map
*R*[*F*^2^ > 2σ(*F*^2^)] = 0.044	Hydrogen site location: inferred from neighbouring sites
*wR*(*F*^2^) = 0.123	H atoms treated by a mixture of independent and constrained refinement
*S* = 0.95	*w* = 1/[σ^2^(*F*_o_^2^) + (0.0725*P*)^2^ + 0.3607*P*] where *P* = (*F*_o_^2^ + 2*F*_c_^2^)/3
4891 reflections	(Δ/σ)_max_ < 0.001
424 parameters	Δρ_max_ = 0.24 e Å^−^^3^
0 restraints	Δρ_min_ = −0.30 e Å^−^^3^

### Special details

**Table:**

Geometry. All s.u.'s (except the s.u. in the dihedral angle between two l.s. planes) are estimated using the full covariance matrix. The cell s.u.'s are taken into account individually in the estimation of s.u.'s in distances, angles and torsion angles; correlations between s.u.'s in cell parameters are only used when they are defined by crystal symmetry. An approximate (isotropic) treatment of cell s.u.'s is used for estimating s.u.'s involving l.s. planes.
Refinement. Refinement of *F*^2^ against ALL reflections. The weighted *R*-factor *wR* and goodness of fit *S* are based on *F*^2^, conventional *R*-factors *R* are based on *F*, with *F* set to zero for negative *F*^2^. The threshold expression of *F*^2^ > σ(*F*^2^) is used only for calculating *R*-factors(gt) *etc*. and is not relevant to the choice of reflections for refinement. *R*-factors based on *F*^2^ are statistically about twice as large as those based on *F*, and *R*- factors based on ALL data will be even larger.

### Fractional atomic coordinates and isotropic or equivalent isotropic displacement parameters (Å^2^)

**Table:**

	*x*	*y*	*z*	*U*_iso_*/*U*_eq_	
C1A	−0.10838 (19)	0.86643 (15)	0.10686 (11)	0.0303 (3)	
O1A	−0.02003 (16)	0.75573 (12)	0.08429 (10)	0.0469 (3)	
C2A	−0.04660 (18)	0.98367 (16)	0.13439 (12)	0.0309 (3)	
N2A	0.12392 (16)	0.97199 (15)	0.15030 (11)	0.0394 (3)	
O21A	0.19522 (16)	1.07010 (15)	0.13867 (13)	0.0601 (4)	
O22A	0.18805 (16)	0.86647 (14)	0.17951 (12)	0.0568 (4)	
C3A	−0.1404 (2)	1.10787 (16)	0.14843 (12)	0.0314 (3)	
H3A	−0.091 (3)	1.179 (2)	0.1651 (16)	0.052 (6)*	
C4A	−0.30793 (18)	1.12289 (14)	0.14285 (11)	0.0284 (3)	
N4A	−0.40990 (18)	1.25160 (13)	0.16554 (10)	0.0350 (3)	
O41A	−0.56396 (15)	1.25745 (13)	0.18252 (10)	0.0474 (3)	
O42A	−0.33873 (18)	1.34906 (12)	0.16819 (12)	0.0566 (4)	
C5A	−0.38244 (19)	1.01553 (15)	0.12063 (11)	0.0288 (3)	
H5A	−0.495 (2)	1.0237 (18)	0.1186 (13)	0.036 (5)*	
C6A	−0.28521 (19)	0.89319 (14)	0.10388 (11)	0.0294 (3)	
N6A	−0.36772 (18)	0.78285 (13)	0.08081 (11)	0.0370 (3)	
O61A	−0.3268 (2)	0.67315 (13)	0.11180 (13)	0.0662 (5)	
O62A	−0.47823 (18)	0.80553 (14)	0.03359 (11)	0.0557 (4)	
C1B	0.29112 (18)	0.24867 (15)	0.41703 (11)	0.0270 (3)	
O1B	0.19899 (13)	0.35763 (11)	0.44572 (8)	0.0353 (3)	
C2B	0.23173 (18)	0.13264 (16)	0.38650 (12)	0.0299 (3)	
N2B	0.05928 (16)	0.14283 (15)	0.37308 (12)	0.0404 (3)	
O21B	−0.00902 (16)	0.04406 (15)	0.38274 (15)	0.0690 (5)	
O22B	−0.00859 (16)	0.24821 (14)	0.34659 (12)	0.0576 (4)	
C3B	0.32794 (19)	0.00995 (16)	0.36639 (12)	0.0315 (3)	
H3B	0.279 (2)	−0.0637 (19)	0.3481 (14)	0.040 (5)*	
C4B	0.49633 (19)	−0.00422 (15)	0.37139 (11)	0.0301 (3)	
N4B	0.59846 (18)	−0.13327 (14)	0.34997 (11)	0.0389 (3)	
O41B	0.74541 (16)	−0.14586 (14)	0.35957 (12)	0.0558 (4)	
O42B	0.53534 (19)	−0.22459 (14)	0.32327 (14)	0.0652 (4)	
C5B	0.56874 (19)	0.10308 (16)	0.39575 (11)	0.0303 (3)	
H5B	0.681 (2)	0.0949 (17)	0.3956 (13)	0.033 (4)*	
C6B	0.46974 (18)	0.22393 (16)	0.41531 (11)	0.0295 (3)	
N6B	0.55497 (17)	0.33587 (15)	0.43280 (12)	0.0414 (4)	
O61B	0.5170 (2)	0.44149 (14)	0.39533 (12)	0.0590 (4)	
O62B	0.66595 (18)	0.31520 (16)	0.47993 (14)	0.0699 (5)	
N11	0.16733 (15)	0.57565 (13)	0.16779 (10)	0.0291 (3)	
H11	0.114 (2)	0.6554 (18)	0.1567 (13)	0.030 (4)*	
C11A	0.2986 (3)	0.5363 (2)	0.07575 (15)	0.0456 (5)	
H11C	0.346 (3)	0.450 (3)	0.0848 (19)	0.071 (7)*	
H11B	0.243 (3)	0.539 (2)	0.024 (2)	0.068 (7)*	
H11A	0.372 (3)	0.600 (3)	0.068 (2)	0.079 (8)*	
C12	0.2433 (2)	0.58588 (17)	0.25660 (13)	0.0348 (4)	
H12B	0.300 (2)	0.498 (2)	0.2664 (14)	0.041 (5)*	
H12A	0.322 (3)	0.647 (2)	0.2416 (16)	0.053 (6)*	
C13	0.1088 (2)	0.63356 (18)	0.34660 (13)	0.0387 (4)	
H13B	0.054 (2)	0.7194 (19)	0.3366 (13)	0.035 (5)*	
H13A	0.157 (2)	0.636 (2)	0.4042 (16)	0.049 (5)*	
N14	−0.02066 (18)	0.54247 (15)	0.36518 (11)	0.0361 (3)	
H14B	−0.100 (3)	0.576 (2)	0.4161 (16)	0.044 (5)*	
H14A	0.031 (3)	0.460 (2)	0.3783 (15)	0.048 (5)*	
C15	−0.0961 (2)	0.52998 (19)	0.27659 (13)	0.0364 (4)	
H15B	−0.153 (2)	0.616 (2)	0.2644 (15)	0.042 (5)*	
H15A	−0.171 (3)	0.471 (2)	0.2906 (15)	0.047 (5)*	
C16	0.0398 (2)	0.48330 (17)	0.18685 (13)	0.0338 (3)	
H16B	0.098 (2)	0.3954 (19)	0.1948 (14)	0.036 (5)*	
H16A	−0.012 (3)	0.481 (2)	0.1283 (17)	0.056 (6)*	

### Atomic displacement parameters (Å^2^)

**Table:**

	*U*^11^	*U*^22^	*U*^33^	*U*^12^	*U*^13^	*U*^23^
C1A	0.0339 (8)	0.0278 (8)	0.0286 (8)	0.0045 (6)	−0.0116 (6)	0.0010 (6)
O1A	0.0507 (7)	0.0349 (6)	0.0543 (8)	0.0144 (5)	−0.0239 (6)	−0.0092 (6)
C2A	0.0257 (7)	0.0361 (8)	0.0310 (8)	−0.0019 (6)	−0.0081 (6)	0.0030 (6)
N2A	0.0275 (7)	0.0450 (8)	0.0462 (9)	−0.0034 (6)	−0.0097 (6)	−0.0010 (7)
O21A	0.0355 (7)	0.0566 (9)	0.0925 (12)	−0.0161 (6)	−0.0166 (7)	0.0067 (8)
O22A	0.0422 (7)	0.0511 (8)	0.0820 (11)	0.0035 (6)	−0.0320 (7)	0.0070 (7)
C3A	0.0342 (8)	0.0290 (8)	0.0324 (8)	−0.0068 (6)	−0.0088 (6)	0.0024 (6)
C4A	0.0311 (7)	0.0244 (7)	0.0284 (8)	0.0010 (6)	−0.0072 (6)	0.0014 (6)
N4A	0.0435 (8)	0.0274 (7)	0.0324 (7)	0.0029 (6)	−0.0099 (6)	−0.0003 (5)
O41A	0.0381 (7)	0.0415 (7)	0.0571 (8)	0.0112 (5)	−0.0094 (6)	−0.0042 (6)
O42A	0.0646 (9)	0.0267 (6)	0.0792 (11)	−0.0052 (6)	−0.0174 (8)	−0.0055 (6)
C5A	0.0280 (7)	0.0307 (8)	0.0287 (8)	−0.0012 (6)	−0.0102 (6)	0.0027 (6)
C6A	0.0357 (8)	0.0260 (7)	0.0289 (8)	−0.0037 (6)	−0.0130 (6)	0.0011 (6)
N6A	0.0453 (8)	0.0307 (7)	0.0387 (8)	−0.0068 (6)	−0.0163 (6)	−0.0020 (6)
O61A	0.0923 (11)	0.0293 (7)	0.0920 (12)	−0.0132 (7)	−0.0508 (10)	0.0087 (7)
O62A	0.0606 (8)	0.0531 (8)	0.0682 (9)	−0.0181 (7)	−0.0407 (8)	0.0056 (7)
C1B	0.0256 (7)	0.0315 (8)	0.0221 (7)	0.0001 (6)	−0.0036 (5)	−0.0009 (6)
O1B	0.0338 (6)	0.0350 (6)	0.0336 (6)	0.0054 (5)	−0.0061 (5)	−0.0059 (5)
C2B	0.0227 (7)	0.0359 (8)	0.0308 (8)	−0.0032 (6)	−0.0055 (6)	0.0006 (6)
N2B	0.0265 (7)	0.0434 (8)	0.0523 (9)	−0.0043 (6)	−0.0108 (6)	−0.0032 (7)
O21B	0.0357 (7)	0.0529 (9)	0.1243 (15)	−0.0158 (6)	−0.0236 (8)	0.0045 (9)
O22B	0.0400 (7)	0.0510 (8)	0.0875 (11)	0.0010 (6)	−0.0315 (7)	0.0076 (7)
C3B	0.0312 (8)	0.0310 (8)	0.0331 (8)	−0.0061 (6)	−0.0070 (6)	−0.0003 (6)
C4B	0.0288 (7)	0.0312 (8)	0.0278 (8)	0.0027 (6)	−0.0050 (6)	−0.0014 (6)
N4B	0.0388 (8)	0.0355 (8)	0.0370 (8)	0.0050 (6)	−0.0031 (6)	−0.0023 (6)
O41B	0.0365 (7)	0.0511 (8)	0.0751 (10)	0.0147 (6)	−0.0155 (6)	−0.0074 (7)
O42B	0.0597 (9)	0.0352 (7)	0.0992 (13)	0.0017 (6)	−0.0186 (8)	−0.0210 (8)
C5B	0.0227 (7)	0.0402 (9)	0.0264 (8)	0.0003 (6)	−0.0053 (6)	−0.0026 (6)
C6B	0.0273 (7)	0.0349 (8)	0.0264 (7)	−0.0051 (6)	−0.0056 (6)	−0.0037 (6)
N6B	0.0318 (7)	0.0453 (9)	0.0462 (9)	−0.0065 (6)	−0.0052 (6)	−0.0166 (7)
O61B	0.0718 (9)	0.0448 (8)	0.0644 (9)	−0.0235 (7)	−0.0133 (8)	0.0015 (7)
O62B	0.0495 (8)	0.0688 (10)	0.0995 (13)	0.0001 (7)	−0.0399 (9)	−0.0343 (9)
N11	0.0286 (6)	0.0248 (6)	0.0310 (7)	0.0034 (5)	−0.0047 (5)	−0.0002 (5)
C11A	0.0464 (10)	0.0415 (11)	0.0383 (10)	0.0077 (9)	0.0055 (8)	0.0011 (8)
C12	0.0280 (8)	0.0350 (9)	0.0423 (9)	−0.0012 (7)	−0.0122 (7)	−0.0009 (7)
C13	0.0431 (9)	0.0357 (9)	0.0386 (9)	0.0020 (7)	−0.0169 (8)	−0.0092 (7)
N14	0.0343 (7)	0.0384 (8)	0.0289 (7)	0.0082 (6)	−0.0010 (6)	−0.0024 (6)
C15	0.0271 (8)	0.0411 (9)	0.0403 (9)	−0.0024 (7)	−0.0073 (7)	0.0020 (7)
C16	0.0376 (8)	0.0309 (8)	0.0342 (9)	−0.0054 (7)	−0.0104 (7)	−0.0033 (7)

### Geometric parameters (Å, °)

**Table:**

C1A—O1A	1.2494 (18)	N4B—O42B	1.220 (2)
C1A—C2A	1.443 (2)	N4B—O41B	1.2271 (19)
C1A—C6A	1.445 (2)	C5B—C6B	1.365 (2)
C2A—C3A	1.372 (2)	C5B—H5B	0.913 (18)
C2A—N2A	1.4470 (19)	C6B—N6B	1.466 (2)
N2A—O21A	1.2233 (19)	N6B—O62B	1.215 (2)
N2A—O22A	1.2283 (19)	N6B—O61B	1.217 (2)
C3A—C4A	1.378 (2)	N11—C16	1.490 (2)
C3A—H3A	0.93 (2)	N11—C12	1.490 (2)
C4A—C5A	1.391 (2)	N11—C11A	1.495 (2)
C4A—N4A	1.4445 (19)	N11—H11	0.892 (18)
N4A—O42A	1.2267 (19)	C11A—H11C	0.93 (3)
N4A—O41A	1.2291 (18)	C11A—H11B	0.92 (3)
C5A—C6A	1.369 (2)	C11A—H11A	0.94 (3)
C5A—H5A	0.917 (19)	C12—C13	1.505 (2)
C6A—N6A	1.4605 (19)	C12—H12B	0.97 (2)
N6A—O62A	1.2144 (18)	C12—H12A	0.96 (2)
N6A—O61A	1.2190 (19)	C13—N14	1.493 (2)
C1B—O1B	1.2612 (18)	C13—H13B	0.946 (19)
C1B—C2B	1.437 (2)	C13—H13A	0.96 (2)
C1B—C6B	1.445 (2)	N14—C15	1.488 (2)
C2B—C3B	1.372 (2)	N14—H14B	0.88 (2)
C2B—N2B	1.4521 (19)	N14—H14A	0.92 (2)
N2B—O21B	1.215 (2)	C15—C16	1.507 (2)
N2B—O22B	1.2238 (19)	C15—H15B	0.96 (2)
C3B—C4B	1.383 (2)	C15—H15A	0.92 (2)
C3B—H3B	0.959 (19)	C16—H16B	0.965 (18)
C4B—C5B	1.390 (2)	C16—H16A	0.99 (2)
C4B—N4B	1.446 (2)		
			
O1A—C1A—C2A	124.85 (14)	C5B—C6B—C1B	124.82 (14)
O1A—C1A—C6A	123.25 (15)	C5B—C6B—N6B	116.40 (13)
C2A—C1A—C6A	111.84 (13)	C1B—C6B—N6B	118.76 (13)
C3A—C2A—C1A	124.24 (13)	O62B—N6B—O61B	123.87 (16)
C3A—C2A—N2A	116.59 (14)	O62B—N6B—C6B	117.52 (16)
C1A—C2A—N2A	119.16 (13)	O61B—N6B—C6B	118.50 (14)
O21A—N2A—O22A	122.68 (14)	C16—N11—C12	110.14 (13)
O21A—N2A—C2A	118.32 (14)	C16—N11—C11A	111.97 (14)
O22A—N2A—C2A	118.91 (14)	C12—N11—C11A	111.84 (14)
C2A—C3A—C4A	119.12 (15)	C16—N11—H11	107.5 (11)
C2A—C3A—H3A	118.9 (13)	C12—N11—H11	109.0 (11)
C4A—C3A—H3A	121.8 (13)	C11A—N11—H11	106.2 (11)
C3A—C4A—C5A	121.40 (14)	N11—C11A—H11C	106.1 (16)
C3A—C4A—N4A	119.07 (14)	N11—C11A—H11B	106.3 (15)
C5A—C4A—N4A	119.46 (13)	H11C—C11A—H11B	110 (2)
O42A—N4A—O41A	123.36 (14)	N11—C11A—H11A	106.9 (17)
O42A—N4A—C4A	118.51 (14)	H11C—C11A—H11A	116 (2)
O41A—N4A—C4A	118.12 (14)	H11B—C11A—H11A	111 (2)
C6A—C5A—C4A	118.49 (14)	N11—C12—C13	110.48 (13)
C6A—C5A—H5A	119.2 (11)	N11—C12—H12B	106.7 (11)
C4A—C5A—H5A	122.3 (11)	C13—C12—H12B	110.9 (11)
C5A—C6A—C1A	124.74 (14)	N11—C12—H12A	107.2 (13)
C5A—C6A—N6A	116.96 (13)	C13—C12—H12A	110.5 (13)
C1A—C6A—N6A	118.30 (13)	H12B—C12—H12A	110.9 (16)
O62A—N6A—O61A	122.69 (14)	N14—C13—C12	110.29 (14)
O62A—N6A—C6A	118.18 (13)	N14—C13—H13B	107.8 (11)
O61A—N6A—C6A	119.09 (13)	C12—C13—H13B	110.5 (11)
O1B—C1B—C2B	124.76 (13)	N14—C13—H13A	108.6 (12)
O1B—C1B—C6B	123.54 (14)	C12—C13—H13A	110.3 (12)
C2B—C1B—C6B	111.65 (13)	H13B—C13—H13A	109.3 (16)
C3B—C2B—C1B	124.68 (13)	C15—N14—C13	111.26 (14)
C3B—C2B—N2B	115.96 (14)	C15—N14—H14B	109.5 (13)
C1B—C2B—N2B	119.34 (13)	C13—N14—H14B	107.5 (13)
O21B—N2B—O22B	122.20 (14)	C15—N14—H14A	108.4 (13)
O21B—N2B—C2B	118.69 (14)	C13—N14—H14A	108.3 (12)
O22B—N2B—C2B	118.96 (14)	H14B—N14—H14A	111.8 (18)
C2B—C3B—C4B	118.77 (15)	N14—C15—C16	110.20 (13)
C2B—C3B—H3B	120.1 (11)	N14—C15—H15B	108.1 (12)
C4B—C3B—H3B	121.1 (11)	C16—C15—H15B	109.3 (12)
C3B—C4B—C5B	121.27 (14)	N14—C15—H15A	108.0 (13)
C3B—C4B—N4B	119.00 (14)	C16—C15—H15A	110.7 (13)
C5B—C4B—N4B	119.73 (13)	H15B—C15—H15A	110.4 (16)
O42B—N4B—O41B	122.89 (14)	N11—C16—C15	110.70 (13)
O42B—N4B—C4B	118.86 (14)	N11—C16—H16B	108.1 (10)
O41B—N4B—C4B	118.24 (14)	C15—C16—H16B	112.1 (11)
C6B—C5B—C4B	118.58 (14)	N11—C16—H16A	108.9 (13)
C6B—C5B—H5B	120.0 (11)	C15—C16—H16A	108.8 (13)
C4B—C5B—H5B	121.3 (11)	H16B—C16—H16A	108.2 (16)
			
O1A—C1A—C2A—C3A	172.55 (16)	C3B—C2B—N2B—O21B	27.4 (2)
C6A—C1A—C2A—C3A	−4.8 (2)	C1B—C2B—N2B—O21B	−153.91 (17)
O1A—C1A—C2A—N2A	−8.5 (2)	C3B—C2B—N2B—O22B	−148.21 (17)
C6A—C1A—C2A—N2A	174.11 (14)	C1B—C2B—N2B—O22B	30.5 (2)
C3A—C2A—N2A—O21A	−26.5 (2)	C1B—C2B—C3B—C4B	−2.8 (2)
C1A—C2A—N2A—O21A	154.53 (16)	N2B—C2B—C3B—C4B	175.80 (14)
C3A—C2A—N2A—O22A	150.03 (16)	C2B—C3B—C4B—C5B	−0.3 (2)
C1A—C2A—N2A—O22A	−29.0 (2)	C2B—C3B—C4B—N4B	−179.82 (14)
C1A—C2A—C3A—C4A	4.6 (2)	C3B—C4B—N4B—O42B	3.6 (2)
N2A—C2A—C3A—C4A	−174.39 (14)	C5B—C4B—N4B—O42B	−175.90 (16)
C2A—C3A—C4A—C5A	−1.8 (2)	C3B—C4B—N4B—O41B	−176.40 (15)
C2A—C3A—C4A—N4A	175.04 (14)	C5B—C4B—N4B—O41B	4.1 (2)
C3A—C4A—N4A—O42A	15.4 (2)	C3B—C4B—C5B—C6B	0.3 (2)
C5A—C4A—N4A—O42A	−167.73 (15)	N4B—C4B—C5B—C6B	179.78 (14)
C3A—C4A—N4A—O41A	−163.68 (14)	C4B—C5B—C6B—C1B	2.9 (2)
C5A—C4A—N4A—O41A	13.2 (2)	C4B—C5B—C6B—N6B	−175.30 (14)
C3A—C4A—C5A—C6A	−0.2 (2)	O1B—C1B—C6B—C5B	172.05 (15)
N4A—C4A—C5A—C6A	−177.01 (14)	C2B—C1B—C6B—C5B	−5.4 (2)
C4A—C5A—C6A—C1A	−0.4 (2)	O1B—C1B—C6B—N6B	−9.8 (2)
C4A—C5A—C6A—N6A	179.97 (13)	C2B—C1B—C6B—N6B	172.77 (14)
O1A—C1A—C6A—C5A	−174.70 (15)	C5B—C6B—N6B—O62B	−38.6 (2)
C2A—C1A—C6A—C5A	2.7 (2)	C1B—C6B—N6B—O62B	143.11 (16)
O1A—C1A—C6A—N6A	4.9 (2)	C5B—C6B—N6B—O61B	137.68 (16)
C2A—C1A—C6A—N6A	−177.70 (13)	C1B—C6B—N6B—O61B	−40.6 (2)
C5A—C6A—N6A—O62A	33.9 (2)	C16—N11—C12—C13	−58.20 (17)
C1A—C6A—N6A—O62A	−145.77 (16)	C11A—N11—C12—C13	176.60 (15)
C5A—C6A—N6A—O61A	−144.12 (17)	N11—C12—C13—N14	57.33 (18)
C1A—C6A—N6A—O61A	36.3 (2)	C12—C13—N14—C15	−56.74 (18)
O1B—C1B—C2B—C3B	−172.05 (15)	C13—N14—C15—C16	56.51 (19)
C6B—C1B—C2B—C3B	5.3 (2)	C12—N11—C16—C15	58.16 (17)
O1B—C1B—C2B—N2B	9.4 (2)	C11A—N11—C16—C15	−176.72 (14)
C6B—C1B—C2B—N2B	−173.25 (14)	N14—C15—C16—N11	−57.15 (19)

### Hydrogen-bond geometry (Å, °)

**Table:**

*D*—H···*A*	*D*—H	H···*A*	*D*···*A*	*D*—H···*A*
N11—H11···O1A	0.892 (18)	1.831 (18)	2.6305 (17)	148.1 (16)
N11—H11···O22A	0.892 (18)	2.356 (18)	2.996 (2)	128.8 (14)
N14—H14B···O1B^i^	0.88 (2)	1.98 (2)	2.8181 (19)	157.3 (17)
N14—H14A···O1B	0.92 (2)	1.99 (2)	2.7962 (18)	146.4 (18)
N14—H14A···O22B	0.92 (2)	2.28 (2)	2.992 (2)	133.9 (16)
C5A—H5A···O21A^ii^	0.917 (19)	2.476 (19)	3.383 (2)	170.3 (16)
C5B—H5B···O21B^iii^	0.913 (18)	2.487 (18)	3.394 (2)	172.3 (15)
C11A—H11C···O41A^iv^	0.93 (3)	2.48 (3)	3.345 (2)	155 (2)
C11A—H11A···O62A^iii^	0.94 (3)	2.57 (3)	3.496 (3)	168 (2)
C12—H12A···O22A	0.96 (2)	2.56 (2)	3.046 (2)	111.6 (15)
C13—H13A···O62B^v^	0.96 (2)	2.46 (2)	3.386 (2)	162.9 (17)

Symmetry codes: (i) −*x*, −*y*+1, −*z*+1; (ii) *x*−1, *y*, *z*; (iii) *x*+1, *y*, *z*; (iv) *x*+1, *y*−1, *z*; (v) −*x*+1, −*y*+1, −*z*+1.
